# Integrative temporal multi-omics reveals uncoupling of transcriptome and proteome during human T cell activation

**DOI:** 10.1038/s41540-024-00346-4

**Published:** 2024-02-28

**Authors:** Harshi Weerakoon, Ahmed Mohamed, Yide Wong, Jinjin Chen, Bhagya Senadheera, Oscar Haigh, Thomas S. Watkins, Stephen Kazakoff, Pamela Mukhopadhyay, Jason Mulvenna, John J. Miles, Michelle M. Hill, Ailin Lepletier

**Affiliations:** 1 QIMR Berghofer Medical Research Institute, Herston, QLD Australia; 2 School of Biomedical Sciences, The University of Queensland, Brisbane, QLD Australia; 3 Faculty of Medicine and Allied Sciences, Rajarata University of Sri Lanka, Saliyapura, Sri Lanka; 4 Australian Institute of Tropical Health and Medicine, James Cook University, Cairns, QLD Australia; 5 Bioinformatics Division, The Walter and Eliza Hall Institute of Medical Research, Melbourne, VIC Australia; 6 School of Computing, University of Colombo, Colombo, Sri Lanka; 7 Faculty of Medicine, The University of Queensland, Brisbane, QLD Australia; 8 Institute for Glycomics, Griffith Univeristy, Gold Coast, QLD Australia

**Keywords:** Immunology, Systems biology

## Abstract

Engagement of the T cell receptor (TCR) triggers molecular reprogramming leading to the acquisition of specialized effector functions by CD4 helper and CD8 cytotoxic T cells. While transcription factors, chemokines, and cytokines are known drivers in this process, the temporal proteomic and transcriptomic changes that regulate different stages of human primary T cell activation remain to be elucidated. Here, we report an integrative temporal proteomic and transcriptomic analysis of primary human CD4 and CD8 T cells following ex vivo stimulation with anti-CD3/CD28 beads, which revealed major transcriptome-proteome uncoupling. The early activation phase in both CD4 and CD8 T cells was associated with transient downregulation of the mRNA transcripts and protein of the central glucose transport GLUT1. In the proliferation phase, CD4 and CD8 T cells became transcriptionally more divergent while their proteome became more similar. In addition to the kinetics of proteome-transcriptome correlation, this study unveils selective transcriptional and translational metabolic reprogramming governing CD4 and CD8 T cell responses to TCR stimulation. This temporal transcriptome/proteome map of human T cell activation provides a reference map exploitable for future discovery of biomarkers and candidates targeting T cell responses.

## Introduction

T cells are key players in adaptive immunity and have a major role in the surveillance against pathogens and tumor cells while maintaining unresponsiveness to self-antigens. The metabolic and protein synthesis machinery that shapes T cell responses is controlled by immune activation. Stimulation of the T cell receptor (TCR) and its co-stimulatory molecule, CD28, initiates a transcriptional program in naïve T cells that leads to activation, expansion, and differentiation into specialized CD4 helper and CD8 cytotoxic T cells^1,2^. The duration of TCR signaling is reported as a key factor in determining the functional qualities of the T cells that develop and their commitment to proliferation^3–5^. In this regard, the time of TCR stimulation necessary to launch the proliferative program for naïve CD4 T cells has been shown to be more than required for CD8 T cells^6,7^.

The extensive reprogramming of activated T cells reflects substantial remodeling of multiple molecular pathways involved in cellular metabolism and protein synthesis increasingly being comprehended due to recent advancements in the fields of proteomics, transcriptomics, metabolomics, and epigenomics^8–12^. Initial genomic and transcriptomic studies laid the baseline for understanding the T cell reprogramming following TCR stimulation but only captured a partial snapshot of this complex process, also involving several protein-protein interactions and protein phosphorylation^13,14^. Our knowledge of changes in the proteome of activated T cells has widened with the application of high throughput proteomic technologies in the field of immunology^8,15–17^. Based on proteomics analysis of mouse primary cells, the competitive proliferative advantage of activated CD8 over CD4 T cells was found to be associated with differences in their intrinsic nutrient transport and biosynthetic capacity^18^.

The caveat of studies based on the traditional single-omics approach is the limitation in providing integrated mRNA-protein data, which offers the opportunity to understand the flow of information that underlies T cell activation and the acquisition of specialized effector functions. Further, many investigations in the fields of T cell immunology and biology originate from observations based on animal experimental models and T cell lines limiting the current knowledge around the immune response of primary human T cells to TCR stimulation^19,20^. As such, mapping the T cell activation cycle at multiple molecular levels is crucial to understanding the complex mechanisms underpinning this process, as well as identifying conditions where TCR activation is disrupted by negative signals that influence the quality of T-cell responses.

In this study, we present an integrative temporal analysis of CD4 and CD8 T cell activation and demonstrate the transcriptome-proteome correlations during TCR-dependent metabolic reprogramming.

## Results

### Uncoupling of T cell proteome and transcriptome following TCR stimulation

**Fig. 1:**
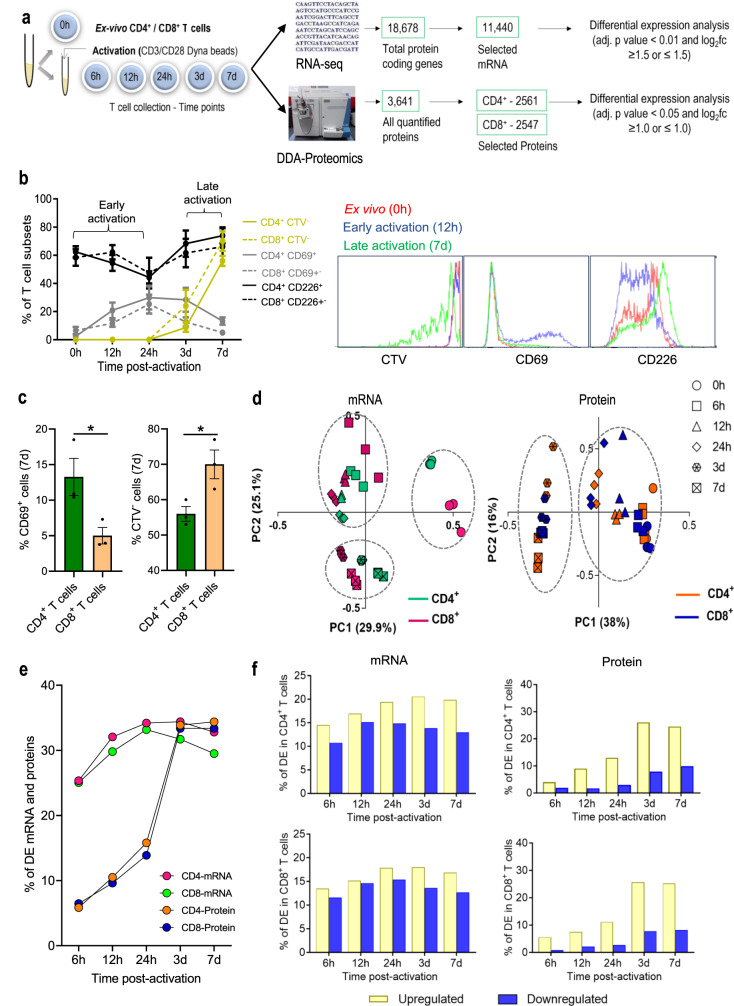
Minor changes to T cell proteome during early stages of activation. **a** Schematic of CD4 and CD8 T cells isolation, TCR stimulation, and parallel transcriptomic and proteomic analysis at 6-time points using RNA sequencing (RNA-seq) and data-dependent acquisition proteomic analysis (DDA-proteomics), respectively. The number of transcripts/proteins pre- and post-quality control is indicated, along with criteria for differential expression (DE) analysis. **b** Characterization of stages of T cell activation using the T cell activation markers, CD69 and CD226, and proliferative T cells (CTV^-^) by flow cytometry; early activation (CD69^high^CD226^low^CTV^+^) and late activation (CD69^high^CD226^low^CTV^-^). **c** Bar graphs show the mean percentage and the standard error of the mean (SEM) error bars of CD69^+^ and CTV^-^ T cells at 7d. Significance was determined using Mann-Whitney rank analysis to compare between CD4 and CD8 T cells. *p < 0.05. **d** Principal component analysis shows the relationship of mRNA and protein data from three biological replicates across different time points. **e** The ratio of mRNA and protein differentially expressed at different time points in relation to their corresponding unstimulated controls (0 h = 1). **f** Bar charts indicate DE genes and proteins in CD4 T cells and CD8 T cells as a percentage of total mRNA/ proteins detected at each time point in relation to unstimulated cells (0 h). yellow: upregulated, blue: downregulated mRNA/ proteins.

Immunology studies often use mRNA transcript level as a surrogate measure of protein abundance, however, several studies in other tissue types have reported limited correlation between the two domains^21–26^. To explore the temporal transcriptomic and proteomic changes following TCR stimulation in both CD4 and CD8 T cells, a time course of ex vivo stimulation with anti-CD3/CD28 beads was conducted using primary T cells purified from the blood of three healthy volunteers and treated as separate replicates. Samples were taken at 0 hour (h), 6 h, 12 h, 24 h, 3 days (d), and 7d (Fig. 1a), using a parallel workflow to generate both transcriptomic and proteomic datasets. These time points were chosen to represent early (up to 24 h) and late phases (3d and 7d) of T cell activation. Prior to RNA sequencing (RNA-seq) and label-free data-dependent acquisition mass spectrometry-based proteomics (DDA-proteomics), the purity of isolated CD4 and CD8 T cells was assessed by fluorescence-labeled flow cytometry (FACS) and monoclonal antibodies to be >90% (Supplementary Fig. 1a, b).

A total of 18,678 protein-coding mRNA and 3531 proteins were identified from a total of 36 samples analyzed (Fig. 1a). Quality control measures computed using RNA-SeQC and MaxQuant output ensured the suitability of the refined transcriptome and proteome datasets, respectively. Based on the similar distribution of mRNA copy numbers (Supplementary Fig. 2a), similar total protein intensities (Supplementary Fig. 2b), as well as <20% missing values for proteins (Supplementary Fig. 2c), 11,440 mRNA transcripts (Supplementary Table 1) and ~2550 proteins (Supplementary Table 2) were selected for differential expression analysis. To characterize the CD4 T cell subtypes at 0 h, cellular deconvolution was conducted on bulk RNA-seq data using CIBERSORTx^27^. The analysis showed the most abundant subtypes being resting memory CD4 T cells (56-78%) and naïve T cells (20-45%), and confirmed lack of contamination from Treg, CD4 activated memory CD4 T cells or CD8 T cells (0%, Supplementary Fig. 1c).

To define the temporal dimensions of T cell responses, we assessed cell proliferation (based on cell trace violet (CTV) dilution) alongside cell activation (based on the detection of surface markers for early (CD69) and late (CD226) phases of activation) (Supplementary Fig. 1d)^28^. In line with previous studies^29^, T cells started to proliferate after 24 h of stimulation (Fig. 1b). Despite the higher levels of CD69 expression, CD4 was slower to proliferate than CD8 T cells (Fig. 1c) (*p*-value ≤ 0.05, Mann-Whitney U-Test). In both CD4 and CD8 T cells, a pronounced CD69 surface expression was elicited between 6 h and 24 h, followed by gradual reduction. Conversely, CD226 expression was transiently downregulated in the first 24 h (Fig. 1b), indicating 24 h as the inflection point between the early and late activation phases in our dataset. This was further supported by principal component (PC) analyses, which revealed that mRNA obtained from unstimulated (0 h), early- or late-activated T cells, formed three well-defined and distinct clusters (Fig. 1d, left). A similar protein clustering pattern was observed between unstimulated and early activated T cells (Fig. 1d, right). There was no obvious difference in T cells between the 3 donors, as evidenced by the close clustering of the three replicates in the PCA plot and dendrogram (Fig. 1d and Supplementary Fig. 3).

Differential expression analysis was conducted by comparing each activation time point to 0 h, revealing the percentage of mRNA or proteins that were differential in each T cell type over time (Fig. 1e, f). As expected, the overall changes in the T cell transcriptome content preceded changes at the protein level. As early as 6 h following TCR stimulation, the expression of ~25% of the transcriptome was significantly changed, in contrast to only ~5% of the proteome, in both T-cell subsets. However, during the proliferation phase (late phase of activation), the fraction of differentially expressed (DE) mRNA transcripts and proteins became almost equal, due to a dramatic increase in proteins but little change in mRNA contents (Fig. 1e).

Together, these data reveal a rapid and drastic T cell transcriptomic response following TCR stimulation, which converts to a refined proteomic response with prolonged stimulation that coincides with proliferation.

### Proteome and transcriptome rewiring coincides with T cell proliferation

**Fig. 2:**
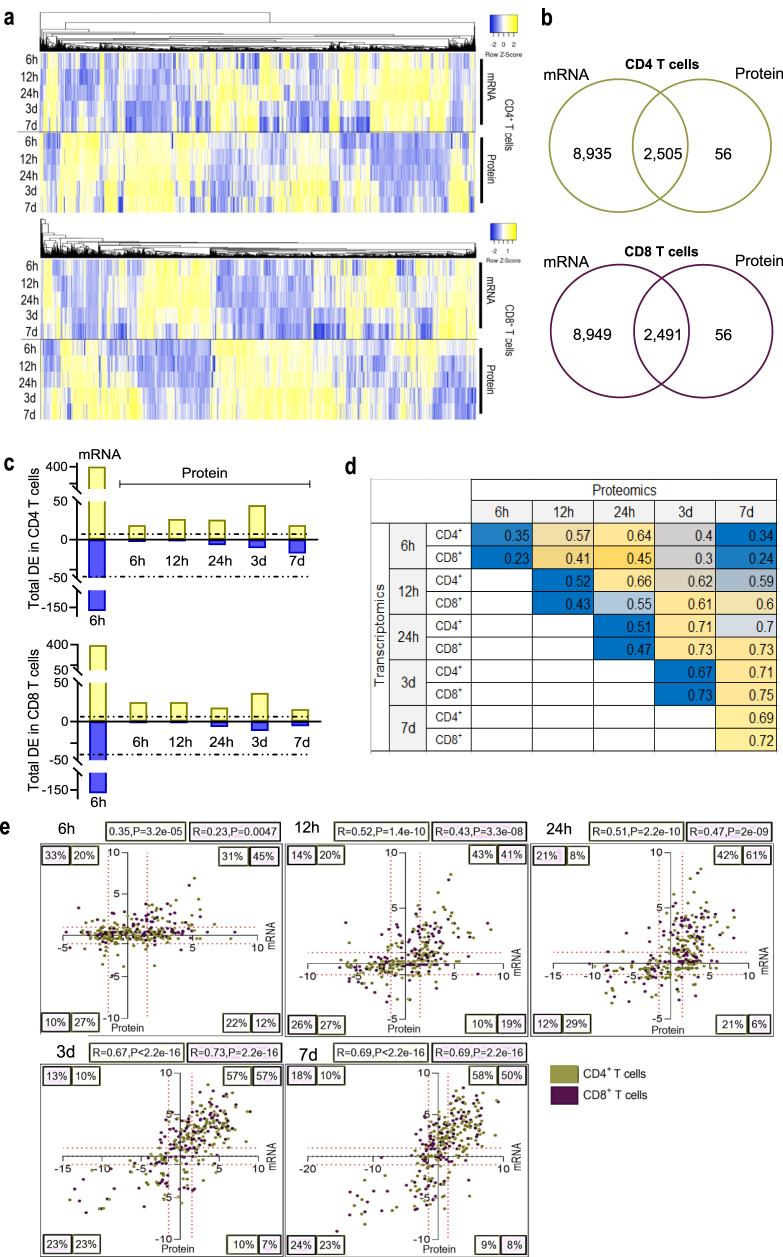
Proteome and transcriptome rewire at late stages of T cell activation. **a** Heatmaps show the expression patterns of commonly quantified mRNA transcripts and proteins in CD4 and CD8 T cells. Yellow: upregulated, blue: downregulated. Row clustering but not column clustering was applied when generating the heatmap. **b** Venn diagrams showing the overlap between quantified mRNA and protein obtained from transcriptomic and proteomic data. **c** DE proteins encoded by mRNA differentially expressed at 6 h following activation. The number of the proteins regulated at each time point is shown. FDR < 0.05. The dotted line represents the total number of proteins upregulated (yellow) or downregulated (blue). **d** Pearson correlation between DE genes and proteins over the entire time course of T-cell activation. Blue to yellow gradient shows low to high correlation values for CD4 and CD8 T cells at each time point. **e** Scatter graph with four quadrants indicates the distribution and correlation between gene and protein expression changes in both CD4 and CD8 T cells at different time points following activation. Each region lists the percentage of T cells falling in each category. mRNA distribution is represented in the “*x*” axis and protein distribution in the “*y*” axis. ‘*R*’ represents the Pearson correlation coefficient.

We hypothesized that the signal propagation required for the conversion of mRNA transcripts into proteins would be temporally regulated during T-cell activation. Supporting this idea, a high discrepancy between the mRNA and protein content was observed in activated CD4 and CD8 T cells (Fig. 2a). Overall, only 20% of mRNA transcripts identified in activated T cells, were quantified at the proteomic level (Fig. 2b), likely due to sensitivity limitations of the DDA-proteomic technology. In view of this limitation, we focused on the DE transcripts, with the rationale that a significant increase in transcript abundance should increase the detectability of the cognate protein. From 570 DE transcripts identified at 6 h, 150 matching proteins were found to be DE during the course of analysis, representing 25 proteins simultaneously modulated at 6 h and over 100 proteins modulated at late phase of activation (Fig. 2c). This expression pattern was common to both CD4 and CD8 T cells and indicates a time delay of at least 3 days for a significant proportion of the mRNA transcripts to be translated into proteins in response to TCR stimulation. Interestingly, a gradual and consistent increase was observed towards the later time points analyzed. While the correlation between proteins and mRNA simultaneously expressed at 6 h was poor (*r* = 0.35 and *r* = 0.23 for CD4 and CD8, respectively), a moderate/strong correlation between mRNA and protein groups was observed at 3d (*r* = 0.67 and *r* = 0.73 for CD4 and CD8 T cells, respectively) and 7d (*r* = 0.69 and *r* = 0.72 for CD4 and CD8, respectively) (Fig. 2d, e).

Thus, a lag between the expression of mRNA transcripts and protein synthesis explains a significant part of the transcriptome-proteome discordance observed in both populations of activated T cells at early phase, allowing for a period of temporal regulation and “omic” rewiring after 3 days of activation.

### TCR stimulation results in proteomic convergence between CD4 and CD8 T cells not mirrored at the mRNA level

**Fig. 3:**
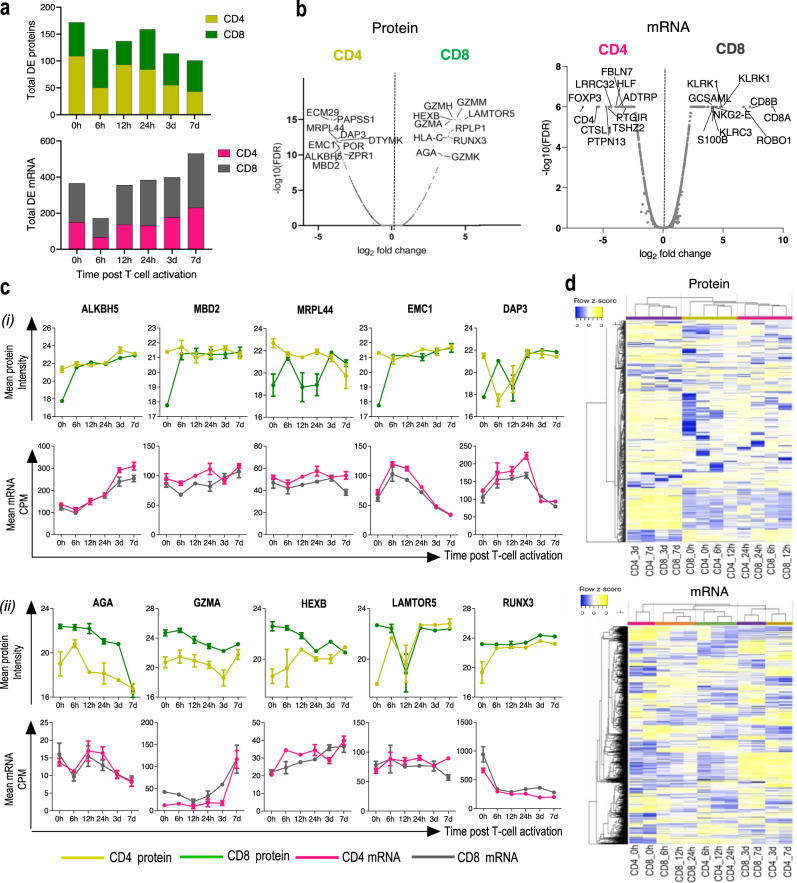
CD4 and CD8 T cells become more divergent following TCR stimulation. **a** Stacked bar graphs showing the total number of proteins and mRNA transcripts DE between CD4 and CD8 T cells at each time point. Columns represent transcripts and proteins overexpressed in each T cell subset. **b** Volcano plots showing proteins mRNA DE between CD4 and CD8 T cells at 0 h. Names of the top 10 overexpressed mRNA and proteins in CD8 and CD4 T cells are indicated. **c** Expression kinetics of proteins upregulated in CD4 (*i*) and CD8 (*ii*) T cells and their corresponding mRNA transcripts. Intensities of each time point are shown as mean and the standard error of the mean (SEM) error bars (*n* = 3). **d** Heatmap shows the relationship of protein/mRNA expression between DE CD4 and CD8 T cells over the time course. mRNA and protein commonly quantified between two T cell subsets were used. Average linkage and Pearson distance measurement were used in column clustering. The clusters of mRNA transcripts and proteins are indicated by distinct colors.

Comparison of mRNA transcript and protein libraries between activated human CD4 and CD8 T cells, from a temporal perspective, has not been previously reported. Therefore, we sought to reveal molecular differences between both T cell subsets by comparing their dynamic transcriptomic and proteomic changes during activation. Among all proteins and transcripts commonly quantified in both subsets, 19% of proteins (487/2544) and 8% of mRNA transcripts (968/11,440) were found to be DE between CD4 and CD8 T cells (Supplementary Table 3). Most of the DE proteins were identified at 0 h (*n* = 172). After 7 days of activation, 42% of DE proteins decreased in relation to 0 h. Overexpressed proteins in CD4 T cells at 0 h included the RNA demethylase (ALKBH5, log2 fold change (log_2_fc) = 3.59), methyl-CpG-binding protein (MBD2, log_2_fc = 3.62) and mitochondrial protein (MRPL44, log_2_fc = 3.59) (Fig. 3b) while overexpressed proteins in CD8 T cells included the regulator complex proteins (LAMTOR5, log_2_fc = 4.57), hexosaminidase subunit beta (HEXB, log_2_fc = 3.99) and lysosomal enzyme (AGA, log_2_fc = 3.38). The transcription regulator Runt-related transcription factor 3 (RUNX3, log_2_fc = 3.75) and distinct profiles of cytotoxic granules (granzymes, GZMM and GZMA), were also among the highest upregulated proteins in CD8 T cells at 0 h (Fig. 3b). Interestingly, during activation, the expression of proteins highly DE at 0 h became more similar between CD4 and CD8 T cells (Fig. 3c, i, top graphs), while the expression of their corresponding transcripts did not significantly change (Fig. 3c, bottom graphs). Despite the overall reduction in the number of DE proteins, CD8 T cells had proteins associated with cytolysis enriched during the entire time course analyzed (Supplementary Fig. 4a).

Among the mRNA transcripts overexpressed in CD8 T cells were the canonical markers CD8A and CD8B, natural killer (NK) cell receptors (KLRK1, KLRC3 and NKG2-E)^30^, and receptors involved in cytolysis (CRTAM and CD160) (Fig. 3b and Supplementary Fig. 4b). CD4 and FOXP3, the transcriptional regulator required for the development of regulatory T cell, were among the overexpressed transcripts in CD4 T cells (Fig. 3b and Supplementary Fig. 4c). In contrast to proteins, the mRNA content became more distinct between activated CD4 and CD8 T cells, and most of the DE mRNA transcripts were identified at 7d (*n* = 530) (Fig. 3a). Supporting lower mRNA discrepancy at 0 h, unsupervised hierarchical analysis comparing expression profiles between CD4 and CD8 T cells revealed that the identified transcripts hierarchically clustered across both cell subsets only at 0 h, showing that following activation the two cell subsets become transcriptionally more distinct (Fig. 3d). At the same time, lower number of clusters were identified from proteomics analysis and plots show that proteins clustered together at 0 h and late activation but not at 6 h and 12 h. This is in accordance with data in Fig. 3a showing that T cells transcriptome becomes more divergent at the same time their proteome becomes more similar.

The significant reduction in the number of DE proteins at late activation indicates the acquisition of similar phenotypic features between CD4 and CD8 T cells coinciding with their proliferative states, which was not captured at the mRNA level.

### Temporal changes in the molecular pathways during distinct phases of T cell responses

**Fig. 4:**
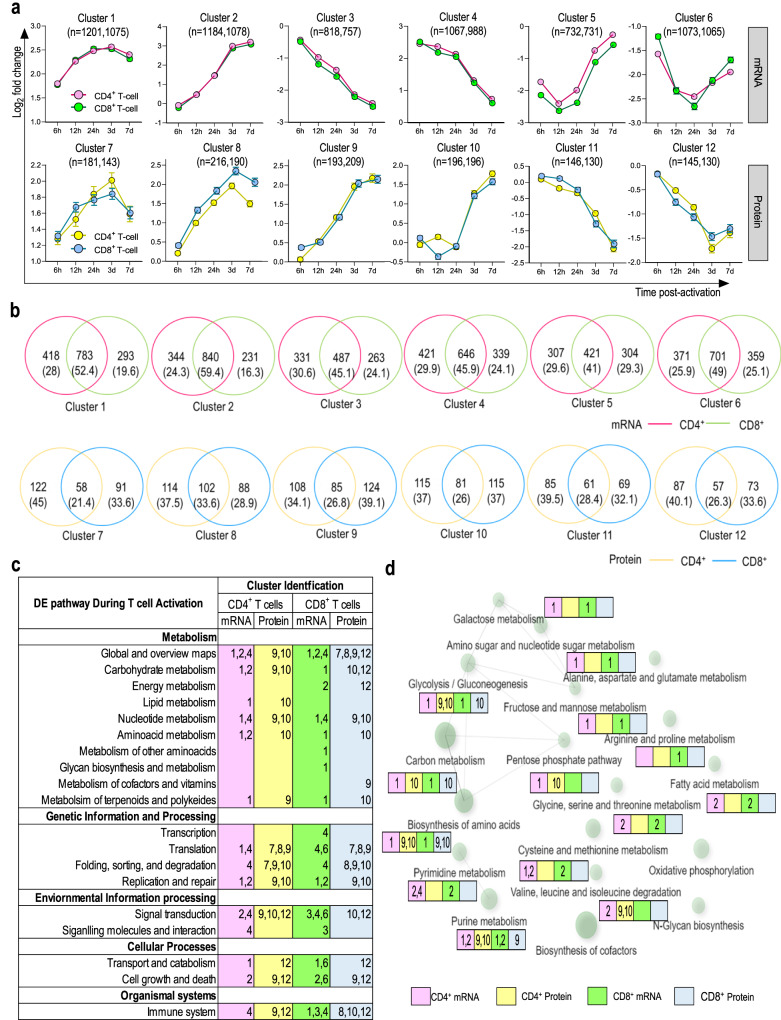
Metabolic signatures in distinct phases of T cell responses. **a** Co-expression cluster of transcriptome and proteome data from CD4 and CD8 T cells. DE mRNA transcripts of each dataset were clustered using mFuzz soft clustering (R package). mRNA or protein intensities at each time point are shown as the mean and the standard error of the mean (SEM) error bars. Number of mRNA/proteins included in each cluster are indicated for CD4 and CD8 T cells, respectively. mRNA transcripts with log2fc > 1.5 or < -1.5 and proteins with log_2_fc ≥ 1.0 or ≤ –1.0 were considered as DE. Data is shown as fold change of mRNA and protein intensities in activated T cells relative to 0 h. **b** Venn diagrams show the overlap of mRNA and protein identified in each cluster between activated CD4 and CD8 T cells. **c** Enriched KEGG pathways (FDR < 0.05) for co-expression clusters (defined in Fig. 4A) of CD4 and CD8 T cell. **d** Molecular interaction, reaction, and relation network showing the relationship of the top first 20 enriched KEGG pathways categorized under ‘metabolism’ (FDR < 0.05). The network was generated using all DE mRNA transcripts and proteins. The size of each node directly correlates with the number of genes included. Edges represent sharing of 20% or more genes between two nodes while the thickness of the edge directly correlates with the number of overlapping genes. The number in each colored box indicates the co-expression clusters in Fig. 4a from where the corresponding mRNA transcript/ protein was enriched.

To elucidate the main cellular pathways supporting CD4 and CD8 T cell activation and proliferation, we used a soft clustering tool to divide mRNA transcripts and proteins that significantly changed following CD3/CD28 stimulation in 12 clusters, defined according to their kinetics of expression (Fig. 4a; Supplementary Fig. 5 and Supplementary Table 4). Although clusters were formed with a similar number of transcripts and proteins representing each T cell subset (Fig. 4a and Supplementary Fig. 5), the overall expression overlap observed between activated CD4 and CD8 T cells identified as part of the same kinetics cluster was intermediate for mRNA transcripts (~50% overlap) and poor for proteins (~25% overlap) (Fig. 4b).

To map the main biological changes underpinning T cell activation, we next conducted a functional pathway enrichment analysis using the Kyoto Encyclopedia of Genes and Genomes (KEGG). Pathways categorized under metabolism, genetic information processing, environmental information processing, cellular processes, and organismal systems were selected for comparative analysis between activated CD4 and CD8 T cells (Fig. 4c). As expected, major changes in metabolic pathways were detected in activated T cells at both protein and transcripts levels. Differences in both mRNA transcripts and proteins governing glycolysis/gluconeogenesis, carbon metabolism, and biosynthesis of amino acids were observed following TCR stimulation. However, changes in the metabolism of essential (methionine and threonine) and non-essential (alanine, aspartate, glutamate, glycine, serine, and cysteine) amino acids were only captured by transcriptomics in both cell subsets, while DE transcripts associated with metabolism of other amino acids and glycan biosynthesis were exclusively detected in CD8 T cells (Fig. 4d). Following TCR stimulation, the protein expression of enzymes related with degradation of fatty acids (ACAT2, ACSL4, ACADVL and HADH) exponentially upregulated in CD4 T cells only (cluster 10) (Fig. 4a, c and Supplementary Table 5). In parallel, proteins associated with energy metabolism and metabolism of cofactors and vitamins were DE solely in CD8 T cells (clusters 9 and 12, respectively) (Fig. 4a, c).

**Fig. 5:**
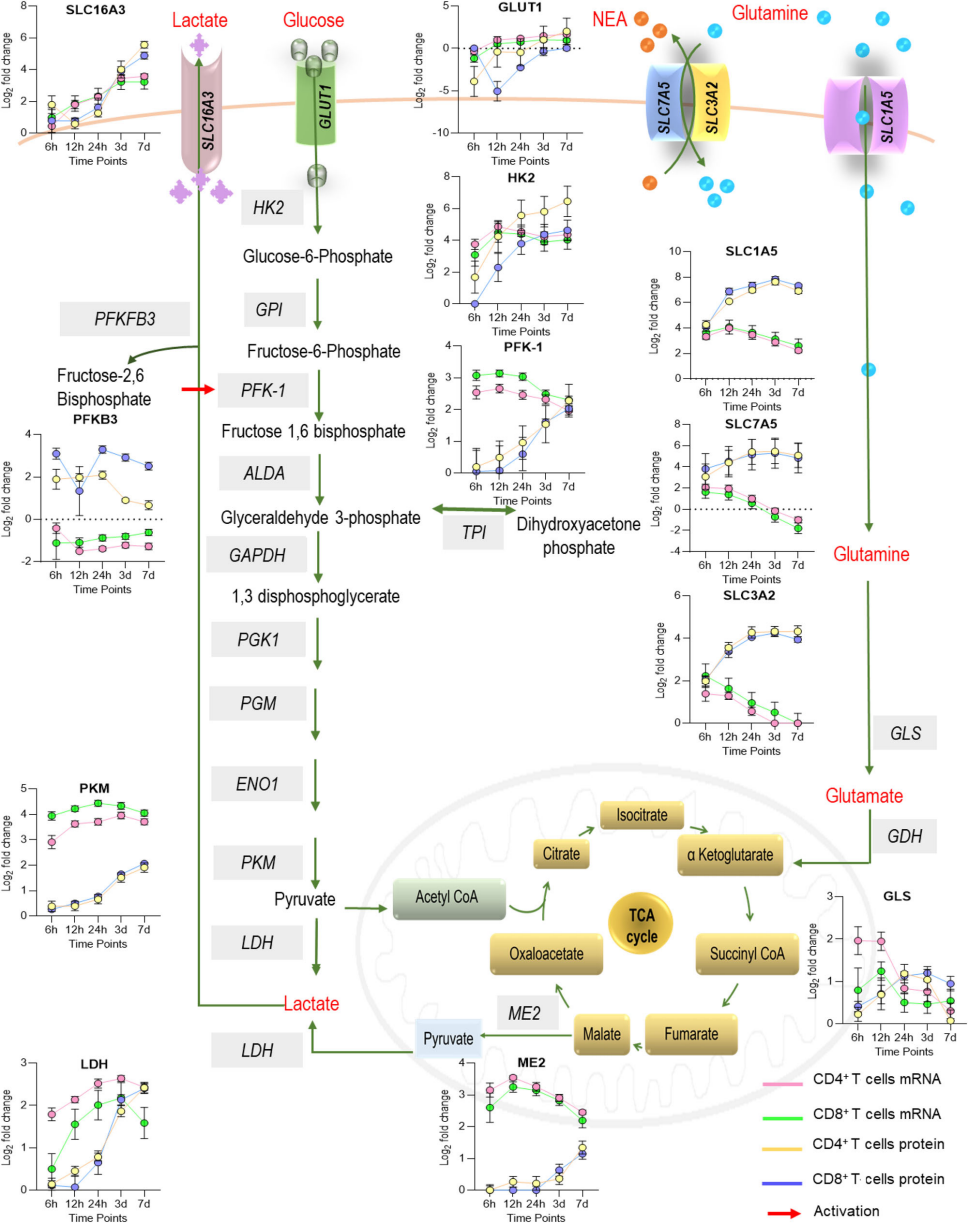
Rewiring of aerobic glycolysis and glutaminolysis results in T cell expansion. T cells utilize glutamine through the glutaminolysis pathway to produce energy during the activation. Graphs show the dynamic protein and gene expression patterns of the main glucose and glutamine transporters and the rate-limiting/ key enzymes of aerobic glycolysis and glutaminolysis during CD4 and CD8 T cell activation (6 h–24 h) and proliferation (3d and 7d). Expressed proteins are named as follows: GLUT-1 (SLC2A1)—the main glucose transporter in T cells, SLC7A5, SLC3A2, and SLC1A5—glutamine transporters, SLC16A3 —lactate transporter, HK2, PFKP, and PKM—the rate-limiting enzymes of glycolysis, PFKFB3—a key allosteric activator of glycolysis, LDH—the enzyme which converts pyruvate to lactate, GLS—the enzyme which converts glutamine to glutamate and ME2—the enzyme which converts malate to pyruvate in the mitochondrial matrix. Data is shown as fold change of mRNA and protein intensities in activated T cells relative to 0 h. mRNA or protein intensities at each time point are shown as the mean and the standard error of the mean (SEM) error bars.

As the transport of nutrients from the surrounding environment is a crucial factor in modulating the molecular mechanisms that lead to activation, we mapped the kinetics of the glucose and amino acid transporters to identify T cell reprogramming following TCR stimulation. Corroborating findings from previous studies^31^, a number of amino acid transporters involved in glutamine uptake (SLC1A5, SLC7A5, and SLC3A2), showed upregulation of their corresponding mRNA and proteins as early as 6 h (Fig. 5 and Supplementary Fig. 6a). Interestingly, upregulated transcripts for these transporters reduced to unstimulated T cell levels after 12 h, while their corresponding proteins exponentially increased during the activation time course in both CD4 and CD8 T cells. Similar expression kinetics were observed for transcripts and proteins representing enzymes in the glutaminolysis pathway (GLS, which convert glutamine into TCA (tricarboxylic acid) cycle metabolites, pyruvate producer ME2, and pyruvate metabolizer LDH) (Fig. 5).

As T cells are known to perform aerobic glycolysis to fulfill the bioenergetic demands of activation^32,33^, we further explored our datasets to identify DE mRNA transcripts and proteins in the glycolysis pathway. Surprisingly, the observed increase in glutamine transport and metabolism was paralleled by a transitory downregulation of protein measures for the main glucose transporter expressed by T cells, GLUT1 (SLC2A1), at 6 h (CD4 T cell) and 12 h (CD8 T cell) following initial stimulation (Fig. 5 and Supplementary Fig. 6b). The results obtained from the high throughput proteomics data were validated by FACS using a fluorescent-labeled monoclonal antibody, which demonstrated downregulation of GLUT1 during the first 12 h in both CD4 and CD8 T cells (Supplementary Fig. 6b). Among the different types of glucose transporters detected by transcriptomics, GLUT1 was the only one detected at the protein level (Supplementary Fig. 6b, c). Interestingly, GLUT1 and GLUT3 mRNA transcripts had similar expression kinetics and progressively increased during activation, showing a more pronounced increase in CD4 T cells. Differently, GLUT6 and GLUT8 were increased in CD8 T cells at 0 h and decreased during activation. Strikingly, in comparison to proteomics, the FACS data revealed a much higher increase in GLUT1 expression after 24 h, maybe reflecting the difference in surface expression captured by FACS versus total GLUT1 captured by proteomics. The decrease in GLUT1 expression was paralleled by transitory downregulation of PFKB3 (a key allosteric activator of glycolysis) protein in CD8 T cells only, whilst PFKB3 mRNA transcript remained downregulated during the entire time course analyzed in both T cell types (Fig. 5 and Supplementary Fig. 6d, e). Protein expression of SLC16A3, a high-affinity transporter capable of exporting lactate and pyruvate in response to the glycolytic influx, transiently dropped at 12 h in CD4 T cells only (Fig. 5). In both CD4 and CD8 T cells, expression of the rate-limiting enzymes of glycolysis, HK2, PKM, and PFK1 increased in the late phase of activation, while their mRNA transcripts were found to be increased as early as 6 h and remained elevated at later time points (Fig. 5).

Altogether, these data demonstrate a transient disconnection between the aerobic glycolysis and glutaminolysis pathways during T cell activation, differently captured across CD4 and CD8 T cells. Such a finding was only possible due to multi-omic analysis and would be overlooked using the single-omics analysis of the T cell transcriptome.

## Discussion

Here we present a study where we compare temporal transcriptomic and proteomic datasets between primary human T cell subsets as a reference for probing the molecular events underpinning different phases of T cell responses. Interrogation of these integrated datasets provided novel insights into the molecular reprogramming kinetics of T cell activation.

Our data indicate a high level of temporal discordance between mRNA transcription and protein expression in T cells following TCR stimulation. Interestingly, by the late phase of activation (after 24 h), we observed concordance between the reprogrammed transcriptome and proteome resulting in proliferation. The correlation between mRNA transcription and protein expression can vary according to the cell type and functional status and a complex discordance in these critical cellular events has been recognized in multiple studies^21–26^. A quantitative proteome and transcriptome mapping of paired healthy human tissues from the Human Protein Atlas project revealed that hundreds of proteins could not be detected for highly expressed mRNA and strong differences were observed between mRNA transcripts and protein quantities within and across tissues^34^. This discordance was partly associated with post-translational modifications of proteins induced by external environmental signals, such as the metabolic flux^35^. We speculate that the discordance observed in this study may be due to insufficient ribosome number/activity to process the rapid transcriptional activation following TCR engagement. This is supported by the observed increase in translation pathway proteins during the early activation phase (Fig. 4c). As changes in other mRNA silencing mechanisms such as RNA decay^36^, degradation by RNases^37^ or sequestration to stress granules^38,39^ were not identified in the present study, we have not specifically evaluated these mechanisms and therefore cannot definitively exclude them at this stage.

T cell subsets have differential requirements for energy and biosynthetic precursors during activation. Therefore, differential programming of key metabolic processes such as glycolysis, fatty acid, and mitochondrial metabolism can direct T cells to particular effector functions^40^. As an example, a metabolic shift from oxidative to glycolytic pathways upon engagement of TCR ensures long-term T cell survival and fuels a fast energy supply for biosynthesis and replication^41^. Our integrated temporal transcriptomics and proteomics design comparing CD4 and CD8 T cells from the same donors uncovered previously uncharacterized selective transcriptional and translational metabolic reprogramming. Following TCR stimulation, the expression of key enzymes in carbohydrate and energy pathways decreased in CD8 T cells. At the same time, CD4 T cells engage enzymes associated with fatty acid degradation to generate acetyl-CoA for the TCA cycle (Fig. 4) and upregulate LAMTOR5 (Fig. 3c). This activates the mTOR pathway, increasing glycolysis and oxidative phosphorylation required for cytokine production. These data show that CD4 T cells have higher mitochondrial respiratory capacities than CD8 T cells. We reveal that among the top 10 proteins upregulated in unstimulated CD4 T cells were the death-associated protein 3 (Dap-3), and a subunit of the endoplasmic reticulum (ER) membrane protein complex (EMC1), which have not been previously characterized in T cells and may play a role in CD4 T cell biology. Interestingly, we found that the protein content of CD4 T cells became gradually more similar to CD8 T cells over time, including the acquisition of cytotoxic functions by CD4 T cells, as characterized by increased levels of GZMA and GZMM. While CD4 T cells with cytotoxic activity able to secrete GZMB and perforin have been observed in various immune responses^42^, a role for GZMM-expressing CD4 T cells is less known. It is possible that the reduction observed in the number of DE proteins between both T-cell subsets is associated with the activation method employed in this study. Even though anti-CD3/CD28 beads generate a more physiologically relevant activation over traditional stimulation methods, such as mitogenic lectins^43^, it is likely that the bulk (polyclonal) response subsequent to this type of activation is leading to a similar developmental program in both T cell subsets, as opposed to conditions where activation is achieved directly through antigen-specific interactions. Supporting this concept, naturally recognized peptides have been shown to produce a different metabolic signature to anti-CD3/CD28 stimulation, associated with their TCR binding affinity^40^.

We report a previously uncharacterized transitory downregulation of GLUT1 during early activation. Although the mechanism controlling transitory GLUT1 downregulation is not demonstrated in this study, GLUT1 surface trafficking is known to be regulated through the co-stimulatory receptor CD28, and tight regulation of this transporter is suggested to be imperative for normal T cell activation^44^. Interestingly, GLUT3 showed a similar kinetics to GLUT1. Despite the high expression of GLUT3 mRNA transcripts, only GLUT1 was detected at the protein level. The low number of identified proteins is partly due to using DDA but also because of the use of the Velos mass spectrometer which gives less hits than new-generation instruments. Despite the decreased expression of the main glucose transporter, glycolysis in activated T cells has been shown to occur independently of the glucose influx^45^. Both lipids and amino acids can be converted into various intermediates of glycolysis and the TCA cycle, allowing them to slip into the cellular respiration pathway through a multitude of side doors including glutaminolysis, the process where glutamine (the most abundant amino acid found in the human body) is converted into mitochondrial TCA cycle intermediates^46,47^. Correlating with the absolute requirement for glutamine to supply carbon and nitrogen to fuel energy necessary for the synthesis of macromolecules in proliferating T cells^48^, our data show an exponential increase in glutamine transporters and glutaminolysis enzymes in activated T cells. We confirm the upregulation of the glutamine transporters, SLC1A5, SLC7A5, and SLC3A2, alongside glutamate synthase (GLS), the key enzyme able to provide glutamine-derived carbons to the TCA cycle^49^. These findings indicate that glutamine may play an important role in fulfilling the early metabolic requirement unleashed by TCR stimulation when intracellular levels of glucose are likely to be low. Supporting the idea of crosstalk between GLUT1 and glutamine transporters in activated T cells similarly to the glucose uptake, transport of glutamine into T cells is dependent on CD28 co-stimulation^44,48^.

The increase in glycolysis and mitochondrial respiration may lead to the accumulation of pyruvate in activated T cells. Accordingly, we evidence upregulation of the mitochondrial malic enzyme (ME2) during late activation, showing that most of the malate originated from the TCA cycle and is likely to be converted to pyruvate rather than oxaloacetate. As pyruvate is rapidly converted into lactic acid in the cell cytoplasm, rather than oxidized in the mitochondrial TCA cycle, a rise in intracellular lactate level will cause premature cell death^33,50^. Our data demonstrate a compensatory mechanism to protect the proliferating CD4 and CD8 T cells from acidosis, mediated by upregulation of the lactate transporter SLC16A3, which allows for efflux of lactate. Interestingly, extracellular lactate correlates with T cell proliferation^51^. Additional research is necessary to investigate the possibilities of lactate recycling for the production of energy, as shown for other human cell types^52^. It is important to emphasize that the current study does not discriminate between CD4 T cells bearing regulatory T (Treg) cells and conventional T helper phenotypes or CD8 T cells bearing MAIT and conventional CD8 T cell phenotypes. Although it is likely that these findings will mostly reflect changes to conventional T cells predominating among PBMC, contamination by unconventional T cells should be taken into consideration when interpreting these findings.

In summary, our study provides integrated temporal transcriptomic and proteomic analysis of molecular events underpinning human primary CD4 and CD8 T cell responses to TCR stimulation. The concordance of mRNA transcript and protein expression changes across multiple time points, revealing the complexity and differences of CD4 and CD8 T cell reprogramming in response to the same generic stimuli. While the current paper focuses on metabolic reprogramming, the matched transcriptomic and proteomic datasets can be used as reference data for human T cell research.

## Methods

### Human CD4 and CD8 T-cell isolation and stimulation

PBMC from healthy controls were freshly isolated from volunteers at QIMR Berghofer for transcriptomic and proteomic studies. Ethics approval was obtained from the human research ethics committee QIMR Berghofer, Brisbane, Queensland, Australia (HREC #P2058). In all cases, PBMC were isolated using a Ficoll‐Paque Plus (Merck, Kenilworth, New Jersey, USA) density gradient centrifugation from blood, and written informed consent was obtained from volunteers.

Human PBMCs isolated from three healthy young adult volunteer blood donors (age 30-35 years, 2 females, 1 male) were further purified using a human pan T-cell isolation kit and magnetically activated cell sorting (MACS) (Miltenyi Biotech, Germany) to isolate unlabeled CD3^+^ T cells. Approximately 30% of total CD3^+^ T cells were purified using a human CD4 T-cell isolation kit while the rest were sorted with a human CD8 T-cell isolation kit (Miltenyi Biotech, Germany) using MACS to obtain untouched CD4 and CD8 T cells, respectively. The sorted cell populations had a purity of over 90%, as assessed by FACS. From each sample, 10^6^ cells were aliquoted for ex vivo proteomics and transcriptomics, respectively. The remainder (~7.5 × 10^6^ cells each) were harvested in Roswell Park Memorial Institute (RPMI) 1640 medium supplemented with 10% Fetal calf serum (Gibco, USA) and 50 units/ml penicillin and 50 μg/ml streptomycin (Gibco, USA) and activated with human T-cell activator anti-CD3/CD28 Dynabeads (Thermo Fisher Scientific, USA) at the bead: cell ratio of 1:1 as per the manufacturer’s instructions. CD8 T-cell cultures were supplemented with 120 IU/ml human recombinant IL-2 (Sigma Aldrich, USA). T cells were aliquoted into five samples with 1.5 × 10^6^ cells in each condition to obtain cells at five different time points (6 h, 12 h, 24 h, 3d, and 7d) and incubated at 37 °C in a humidified, 5% CO_2_ incubator. The culture medium was changed on day 4 of post-activation. At each time point, aliquots of activated T cells were collected washed three times with phosphate-buffered saline (PBS), and stored for batch proteomics/transcriptomics processing. T cells for proteomics were stored at –80 °C. For transcriptomics, cells were lysed in 400 μl of cold TRIzol before storage at -80 °C until RNA processing. At 0 h, 10^6 ^T cells contained ~35–40 μg of Protein (Pierce BCA protein quantification kit, Thermo Fisher Scientific, USA) and ~900–1000 ng of total RNA (qubit fluorometer, Invitrogen, Thermo Fisher Scientific, USA). Samples from the three donors were treated as separate replicates and not pooled for downstream assays.

### Monitoring the in vitro T-cell activation process

T-cell activation and proliferation were monitored using T-cell activation markers and a T-cell proliferation assay. In parallel to the main experiment, a sample of CellTraceTM Violet (CTV) (1:1000, cat. C34557, Thermo Fisher Scientific, USA) stained T cells (CD4 and CD8) from each donor was performed as per the protocol given by the manufacturer. At each time point a sample of cells was stained with CD69-PE-cy7 (1:200, cat. 557745, BD biosciences, USA), CD226-FITC (1:300, cat. 559788, BD Biosciences, USA) GLUT-1-(1:200, cat. 566580, BD Biosciences, USA) along with CD3-APCe780 (1:300, cat. 47-0032-82, eBioscience, USA), CD4-BV711 (1:400, cat. 563033, BD Bioscience, USA) and CD8-PerCP/Cy5.5 (1:200, cat. 344710, Biolegend, USA). Samples were analyzed using FACS to determine dynamic expression changes. The percentage of CTV- cells were analyzed to determine the T-cell proliferation rate at different time points.

### RNA extraction, mRNA library generation, and next-generation sequencing (NGS)

Total RNA was extracted using TRIzol (Thermo Fisher Scientific, USA) phase separation, following the protocol given by the manufacturer. Ultrapure glycogen (Thermo Fisher Scientific, USA) was used to precipitate total RNA. The quality and quantity of the extracted RNA were analyzed using a qubit fluorometer (Invitrogen, Thermo Fisher Scientific, USA) and Agilent 2100 bioanalyzer (Agilent Technologies, USA), respectively. and a RIN score of over 8.00 was confirmed for all the samples (*n* = 36). In each sample, 300 ng of total RNA was aliquoted and mRNA libraries were prepared using a TruSeq stranded mRNA library preparation kit (Illumina, USA). After quality and quantity assessment of the generated libraries, next-generation sequencing (NGS) was performed using NextSeq 500/550 high output v2 kit (150 cycles) (Illumina, USA) to obtain 800 × 10^6^ paired reads per pool (50 ×10^6^ paired reads per sample). Library generation and NGS were performed at the analytical facility, QIMR Berghofer.

### Proteomic sample preparation and liquid chromatography–mass spectrometry (LC-MS/MS) data acquisition

T cells (1 ×1 0^6^) were lysed in 2% SDS in 100 mM TEAB in the presence of a protease inhibitor cocktail. After assessing the protein quantity using Pierce BCA protein quantification kit (Thermo Fisher Scientific, USA), ~ 20 μg from each cell lysate was separated and 200 ng of ovalbumin was added as the internal standard. These samples were reduced, alkylated, and digested using trypsin following the method samples as previously described^15^ to obtain the peptides. After desalting, peptides were quantified using microBCA (Thermo Fisher Scientific, USA) protein assay to aliquot 1 μg from each sample for MS analysis and were resuspended in MS grade water with 2% acetonitrile, 0.1% formic acid (v/v) to obtain the final volume of 10 μl. These samples were injected into a Protecol C18 trap column in Prominence Nano (Shimadzu, Japan) LC system to separate the ions in a Protecol C18 (200 Å, 3 μm particle size, 150 mm × 150 μm) column at a flow rate of 1 μl/min over 180 min linear gradient. Solvent A (0.1% formic acid) and solvent B (100% acetonitrile and 0.1% formic acid) were used for the mobile phase. Peptides were eluted in three consecutive linear gradients: 5–10% solvent B over 5 min, 10–27% solvent B over 147 min, and 27–40% solvent B over 10 min. Finally, the column was cleaned using 40% to 95% solvent B for 10 min. Chromeleon software (version 6.8, Dionex) embedded in Xcalibur software (version 3.0.63, Thermo Fisher Scientific) was used in the nano LC system. Peptides ionized by the nanospray (Thermo Fisher Scientific, USA) ion source (ion spray voltage –1.75 V, heating temperature 285 °C) were analyzed using a Velos Pro Orbitrap mass spectrometer (Thermo Fisher Scientific, USA). In DDA-MS, the MS was controlled and operated in the “top speed” mode using the Xcalibur software to obtain MS1 and MS2 spectral data for peptide ions with charge status between +2 to +4 at 1.96 s window time.

### Transcriptomic data analysis

Sequence reads were trimmed for adapter sequences using Cutadapt (version 1.11)^53^ and aligned using STAR (version 2.5.2a)^54^. to the GRCh37 assembly with the gene, transcript, and exon features of Ensembl (release 89) gene model. Quality control metrics were computed using RNA-SeQC (version 1.1.8)^55^, while gene expression was estimated using RSEM (version 1.2.30)^56^. Both counts per million (CPM) and trimmed mean of *M*-values (TMM) methods were used to normalize the gene expression data and differential expression analysis was carried out using edgeR (R package)^57^. mRNA with log2fc > 1.5 or < -1.5 at adj. *p*-value of <0.01 were considered as differentially expressed (DE), up- and downregulated genes, respectively.

To estimate the proportions of T-cell subsets cellular deconvolution analysis was conducted using CIBERSORTx^27^. The reference gene sets (93 genes with specific high expression in CD4 and CD8 T cells, shown in Supplementary Fig. 1c) were extracted from existing signature matrix data ‘LM22’^58^ utilizing a density-based clustering method HDBSCAN^59^. LM22 is a microarray-derived signature matrix with 547 genes differentially expressed across 22 human hematopoietic cell subsets in bulk tissues, including tumors. ‘B batch correction’ was applied to correct platform effects between microarray and RNA-seq data.

### Proteomic data analysis

After inspecting the quality of generated DDA-MS data using RawMeat (Vast Scientific), raw files were analyzed using MaxQuant software^60^ against UniProt reviewed human proteome database containing 20,242 entries (downloaded on 25th October 2017), UniProt chicken ovalbumin (UniProt ID—P01012) fasta file and the list of common MS contaminants included in the software. maxLFQ^61^ was used to obtain normalized protein intensity data. The peptide and protein data quality filter was set at 1% FDR. For statistical analysis, proteins with only 1 unique or razor peptide or *m*-score value less than 5 were excluded. Proteins with log_2_fc ≥ 1.0 or ≤ –1.0 at a *q*-value of ≤0.05 were considered as statistically up- and downregulated, respectively.

### Identification of differentially expressed (DE) mRNA transcripts and proteins

To obtain statistical significance, mRNA transcripts, and protein intensities (log2 transformed) of activated T cells at different time points were compared against unstimulated samples (0 h) using multiple t-tests with false discovery determination by the two-stage linear step-up procedure of Benjamini, Krieger, and Yekutieli (*q-*value)^62^. The three donors were considered as biological replicates for DE analysis and log2 fold-change was calculated using their mean values. The percentage of DE mRNA and protein was calculated as the number of DE genes/total number of quantified genes) x 100. In this way, one set of DE genes/proteins was identified for each time point.

### Correlation analysis, clustering, and gene enrichment analysis of differentially expressed mRNA and protein

Commonly quantified mRNA and protein were selected by mapping UniProt IDs of proteomic data with the Ensemble IDs using UniProt Retrieve/ID mapping. Using log_2_fc values Pearson correlation between DE mRNA transcripts and their corresponding protein was calculated for different time points. Correlation coefficient values ±(1.00–0.70) were considered as a strong correlation while ± (0.69–0.40) and ± (0.39–0.10) were taken as moderate and weak respectively^63^. To identify the co-expression clusters over the course of activation, mRNA or protein significant in at least one time point were filtered and clustered using the Mfuzz soft clustering method (R package)^64^. KEGG pathways^65^ enriched (FDR ≤ 0.05) by the genes represented by each cluster were identified using String: functional protein network analysis version 11.5^66^ and ShinyGO 0.76^67^. Word clouds were generated using the online tool (https://www.wordclouds.com, accessed December 2021). Bioinformatics analysis and graph generation were done using R Studio^68^ and GraphPad Prism (version 9.2.0 for Windows, GraphPad Software, San Diego, California USA).

### Reporting summary

Further information on research design is available in the Nature Research Reporting Summary linked to this article.

### Supplementary information

Supplementary Tables 1-6

Reporting summary

Supplementary Figures

## Data Availability

The mass spectrometry proteomics data have been deposited on the ProteomeXchange Consortium via the PRIDE partner repository with the dataset identifier PXD038810. The RNA-seq raw sequence data are not publicly available because participants did not give consent for the data to be publicly released. The RNA-seq gene count data is given in Supplementary Table 6.
