# Supplementary material for: Integrative temporal multi-omics reveals uncoupling of transcriptome and proteome during human T cell activation
Source: NPJ Syst Biol Appl. 2024 Feb 28;10:21. doi: 10.1038/s41540-024-00346-4 (PMC10901835; doi:10.1038/s41540-024-00346-4)
Supplement: Supplementary file 2 — Reporting summary [file 41540_2024_346_MOESM2_ESM.pdf]

nature portfolio

Corresponding author(s): Dr. Ailin Lepletier
NPJSBA-01159R

Last updated by author(s): 2023/10/17

Reporting Summary

Nature Portfolio wishes to improve the reproducibility of the work that we publish. This form provides structure for consistency and transparency in reporting. For further information on Nature Portfolio policies, see our Editorial Policies and the Editorial Policy Checklist.

Statistics

For all statistical analyses, confirm that the following items are present in the figure legend, table legend, main text, or Methods section.

n/a	Confirmed	
☐	☒	The exact sample size (*n*) for each experimental group/condition, given as a discrete number and unit of measurement
☐	☒	A statement on whether measurements were taken from distinct samples or whether the same sample was measured repeatedly
☐	☒	The statistical test(s) used AND whether they are one- or two-sided<br>*Only common tests should be described solely by name; describe more complex techniques in the Methods section.*
☐	☒	A description of all covariates tested
☐	☒	A description of any assumptions or corrections, such as tests of normality and adjustment for multiple comparisons
☐	☒	A full description of the statistical parameters including central tendency (e.g. means) or other basic estimates (e.g. regression coefficient) AND variation (e.g. standard deviation) or associated estimates of uncertainty (e.g. confidence intervals)
☒	☐	For null hypothesis testing, the test statistic (e.g. *F*, *t*, *r*) with confidence intervals, effect sizes, degrees of freedom and *P* value noted<br>*Give P values as exact values whenever suitable.*
☒	☐	For Bayesian analysis, information on the choice of priors and Markov chain Monte Carlo settings
☐	☒	For hierarchical and complex designs, identification of the appropriate level for tests and full reporting of outcomes
☒	☐	Estimates of effect sizes (e.g. Cohen's *d*, Pearson's *r*), indicating how they were calculated

Our web collection on statistics for biologists contains articles on many of the points above.

Software and code

Policy information about availability of computer code

Data collection	Publicly available software or the software incorporated to the instruments were used for proteomic and transcriptomic data collection and analysis.<br>Proteomic data: Chromeleon software (version 6.8, Dionex) embedded in Xcalibur software (version 3.0.63, Thermo Fisher Scientific) was used in the nano LC system<br>Transcriptomic data: Illumina nextseq 550 platform (Illumina, USA)
Data analysis	Proetomic data: RawMeat (Vast Scientific), MaxQuant software, maxLFQ, , R Studio, GraphPad Prism<br>Trancriptomic data: Cutadapt (version 1.11), STAR (version 2.5.2a), RNA-SeQC (version 1.1.8) RSEM (version 1.2.30), edgeR (R package), GraphPad Prism

For manuscripts utilizing custom algorithms or software that are central to the research but not yet described in published literature, software must be made available to editors and reviewers. We strongly encourage code deposition in a community repository (e.g. GitHub). See the Nature Portfolio guidelines for submitting code & software for further information.

Data

Policy information about availability of data

All manuscripts must include a data availability statement. This statement should provide the following information, where applicable:

- Accession codes, unique identifiers, or web links for publicly available datasets
- A description of any restrictions on data availability
- For clinical datasets or third party data, please ensure that the statement adheres to our policy

Mass spectrometry proteomic data have been deposited on the ProteomeXchange Consortium via the PRIDE partner repository with the dataset identifier PXD038810. The RNAseq raw sequence data are not publicly available because participants did not give consent for the data to be publicly released. The RNAseq genecount data is given in Supplementary Table 6.

Research involving human participants, their data, or biological material

Policy information about studies with human participants or human data. See also policy information about sex, gender (identity/presentation), and sexual orientation and race, ethnicity and racism.

Reporting on sex and gender	We used blood samples obtained from three biological replicates representing 2 females and 1 male (self reported). We do not report individual data and the biological replicates are identified as Replicate 1, 2 and 3. Further, gender was not considered in the data analysis as we did not observe a significant difference female and male donors.
Reporting on race, ethnicity, or other socially relevant groupings	We do not report race, ethnicity or any social group
Population characteristics	Healthy adults aged between 30-35 years
Recruitment	Blood samples were obtained from the volunteer blood donors at QMIR Berghofer, Brisbane Australia after obtaining informed written consent
Ethics oversight	Ethics approval was obtained from the human research ethics committee, QIMR Berghofer, Brisbane, Australia (HREC#P2058)

Note that full information on the approval of the study protocol must also be provided in the manuscript.

Field-specific reporting

Please select the one below that is the best fit for your research. If you are not sure, read the appropriate sections before making your selection.

☒ Life sciences ☐ Behavioural & social sciences ☐ Ecological, evolutionary & environmental sciences

For a reference copy of the document with all sections, see nature.com/documents/nr-reporting-summary-flat.pdf

Life sciences study design

All studies must disclose on these points even when the disclosure is negative.

Sample size	CD4 and CD8 T cells isolated from 3 participants were used
Data exclusions	Peptide and protein data quality filter was set at 1% FDR. For statistical analysis, proteins with only 1 unique or razor peptide or m-score value less than 5 were excluded. Quality control metrics of transcriptomic data were computed using RNA-SeQC (version 1.1.8).
Replication	3 biological replicates
Randomization	We isolated CD4 and CD8 T cells from 3 biological replicates randomly selected. After separating samples to assess ex vivo (0h) proteome and transcriptome, CD4 or CD8 T cells were seeded onto distinct culture flasks for activation with anti-CD3/CD28 beads. Samples were randomized during the subsequent steps followed to acquire proteomic and transcriptomic data.
Blinding	Blinding was not required in the study as all biological replicates have been treated in the same way.

Reporting for specific materials, systems and methods

We require information from authors about some types of materials, experimental systems and methods used in many studies. Here, indicate whether each material, system or method listed is relevant to your study. If you are not sure if a list item applies to your research, read the appropriate section before selecting a response.

Materials & experimental systems

n/a	Involved in the study
☐	☒ Antibodies
☒	☐ Eukaryotic cell lines
☒	☐ Palaeontology and archaeology
☒	☐ Animals and other organisms
☒	☐ Clinical data
☒	☐ Dual use research of concern
☒	☐ Plants

Methods

n/a	Involved in the study
☒	☐ ChIP-seq
☐	☒ Flow cytometry
☒	☐ MRI-based neuroimaging

Antibodies

Antibodies used

1. Human antiCD3 monoclonal antibody -APCefluro-780 (SK7, eBioscience, Thermo fisher scientific,USA), 2. Human antiCD4 monoclonal antibody-BV711 (SK3, BD Biosciences, USA) 3. Human antiCD8 monoclonal antibody-PerCp/cy5.5 (SK1, Bioledgend, USA), 4. Human antiCD69 monoclonal antibody-PE cy7 (FN50, BD Biosciences, USA) 5. Human antiCD226 monoclonal antibody-BV711 (DX11, BD Biosciences, USA), 6. Human antiGLUT1 antibody-AF647 (EPR 3915, Abcam, Australia)

Validation

Describe the validation of each primary antibody for the species and application, noting any validation statements on the manufacturer's website, relevant citations, antibody profiles in online databases, or data provided in the manuscript.

Plants

Seed stocks

Report on the source of all seed stocks or other plant material used. If applicable, state the seed stock centre and catalogue number. If plant specimens were collected from the field, describe the collection location, date and sampling procedures.

Novel plant genotypes

Describe the methods by which all novel plant genotypes were produced. This includes those generated by transgenic approaches, gene editing, chemical/radiation-based mutagenesis and hybridization. For transgenic lines, describe the transformation method, the number of independent lines analyzed and the generation upon which experiments were performed. For gene-edited lines, describe the editor used, the endogenous sequence targeted for editing, the targeting guide RNA sequence (if applicable) and how the editor was applied.

Authentication

Describe any authentication procedures for each seed stock used or novel genotype generated. Describe any experiments used to assess the effect of a mutation and, where applicable, how potential secondary effects (e.g. second site T-DNA insertions, mosiacism, off-target gene editing) were examined.

Flow Cytometry

Plots

Confirm that:

☒ The axis labels state the marker and fluorochrome used (e.g. CD4-FITC).

☒ The axis scales are clearly visible. Include numbers along axes only for bottom left plot of group (a 'group' is an analysis of identical markers).

☒ All plots are contour plots with outliers or pseudocolor plots.

☒ A numerical value for number of cells or percentage (with statistics) is provided.

Methodology

Sample preparation

Prior to sample staining, concentrations of each fluorescent labelled mAb was optimized to the cell number. For surface staining, cells were washed twice with R10 at 340xg for 5 minutes, and incubated at 40C for 20 minutes in the dark with antibody mixes prepared in FACS buffer (2% FCS in PBS). For antibody staining ~1 x 106 cells were incubated in 50 µl FACS buffer containing antibody mixes. Unstained, single colour stained, isotype stained or fluorescence minus one (FMO) stained cells were included as controls where required. At the end of the incubation period, samples were washed and resuspended in the appropriate volume of FACS buffer for acquisition on a flow cytometer.

Instrument

BD LSRFortessa with four or five lasers installed (BD Biosciences, USA)

Software

Flowjo (Flowjo, USA)

Cell population abundance

The purity of sorted CD4 and CD8 T cells was assessed by fluorescence-labeled flow cytometry (FACS) and monoclonal antibodies to be over 90%

Gating strategy	Lymphocytes were the initial gate on the FSC, SSC plot followed by viable CD3+ cells.

☒ Tick this box to confirm that a figure exemplifying the gating strategy is provided in the Supplementary Information.
