# Supplementary material for: Integrative temporal multi-omics reveals uncoupling of transcriptome and proteome during human T cell activation
Source: NPJ Syst Biol Appl. 2024 Feb 28;10:21. doi: 10.1038/s41540-024-00346-4 (PMC10901835; doi:10.1038/s41540-024-00346-4)
Supplement: Supplementary file 3 — Supplementary Figures [file 41540_2024_346_MOESM3_ESM.pdf]

Supplementary Figure 1

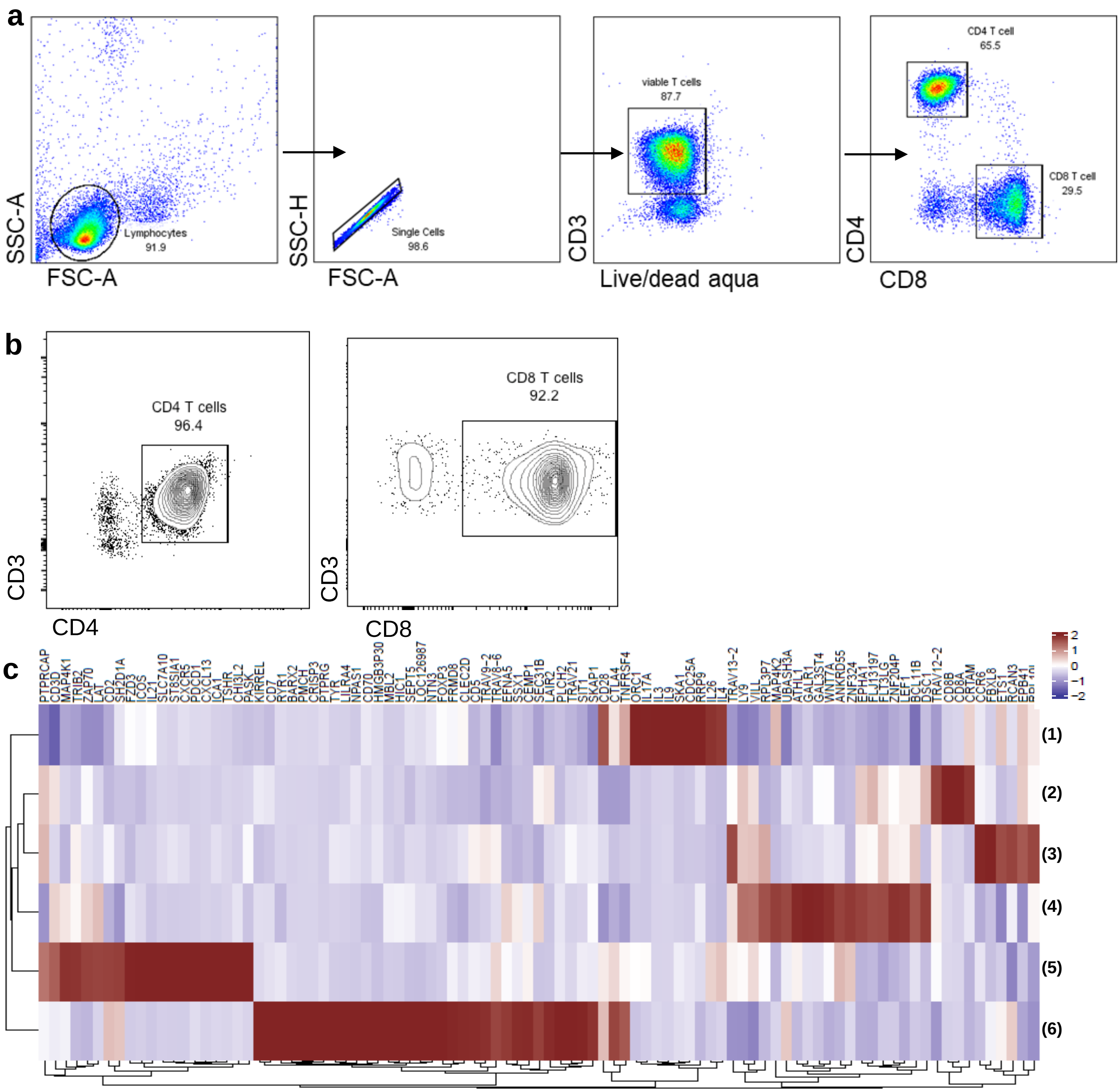

Replicate	CD4 mem activated (1)	CD8 (2)	CD4 mem resting (3)	CD4 naïve (4)	Follicular helper (5)	Tregs (6)	Correlation	RMSE
CD4_0h_1	0	0	0.557905	0.442095	0	0	0.73988	0.682042
CD4_0h_2	0	0	0.789285	0.204906	0.00581[illegible]	0	0.792337	0.612061
CD4_0h_3	0	0	0.68284	0.31374	0.003421	0	0.791249	0.61359

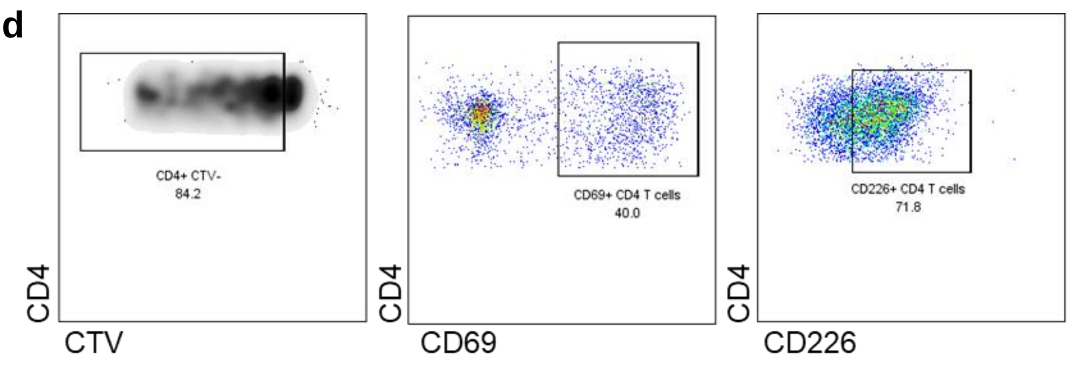

a. Flow cytometry gating strategy for CD4 and CD8 T cell sorting (0h).
b. Scatter plots obtained from FACS analysis evidencing the purity of sorted CD4 and CD8 T cells.
c. Heatmap of scaled expression of 93 selected genes extracted from LM22 to identify six T cell subsets within sorted CD4 T cells at 0h. Upregulated genes are shown in red and downregulated genes are shown in blue for each T cells subset identified as (1) CD4 memory activated T cell, (2) CD8 T cell, (3) CD4 memory resting T cell, (4) CD4 naïve T cell, (5) Follicular helper T cell (6) Regulatory T cell. Table show proportions, correlation and Root-mean-square error (RMSE) of identified T cell subsets.
d. Flow cytometry strategy for analysis of CD4 T cells $CTV^{-/ow}$ (d7), $CD69^{+}$ (d3) and $CD226^{+}$ (d3).

Supplementary Figure 2

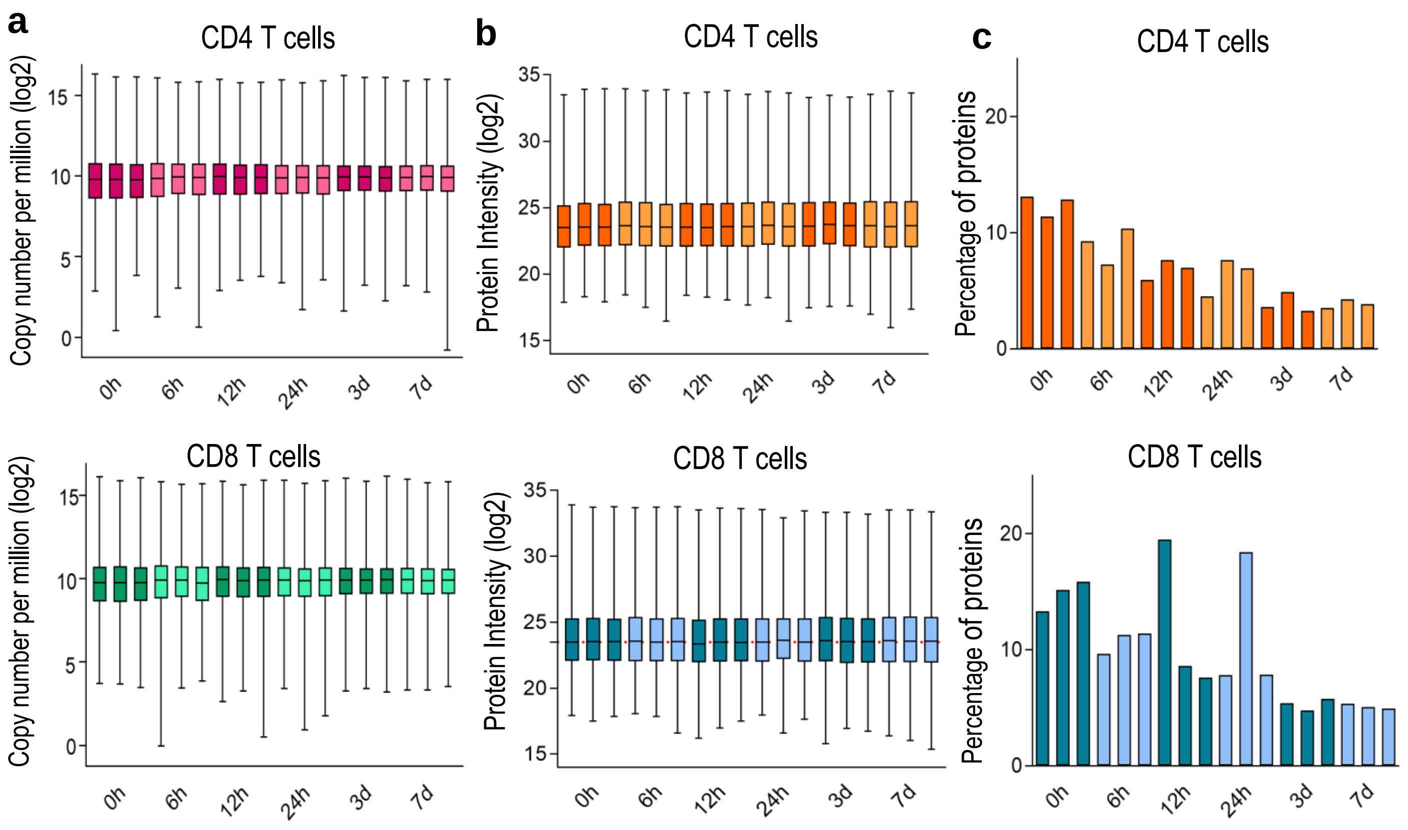

a. Box plots showing the mRNA copy number distribution in CD4 and CD8 T cell samples (box plots are median, 2nd and 3rd quartiles, whiskers are 95% CI)

b. Distribution of protein intensity data in CD4 and CD8 T cell samples (box plots are median, 2nd and 3rd quartiles, whiskers are 95% CI)

c. Percentage of proteins with missing intensity values in different CD4 and CD8 T cell samples.

Supplementary Figure 3

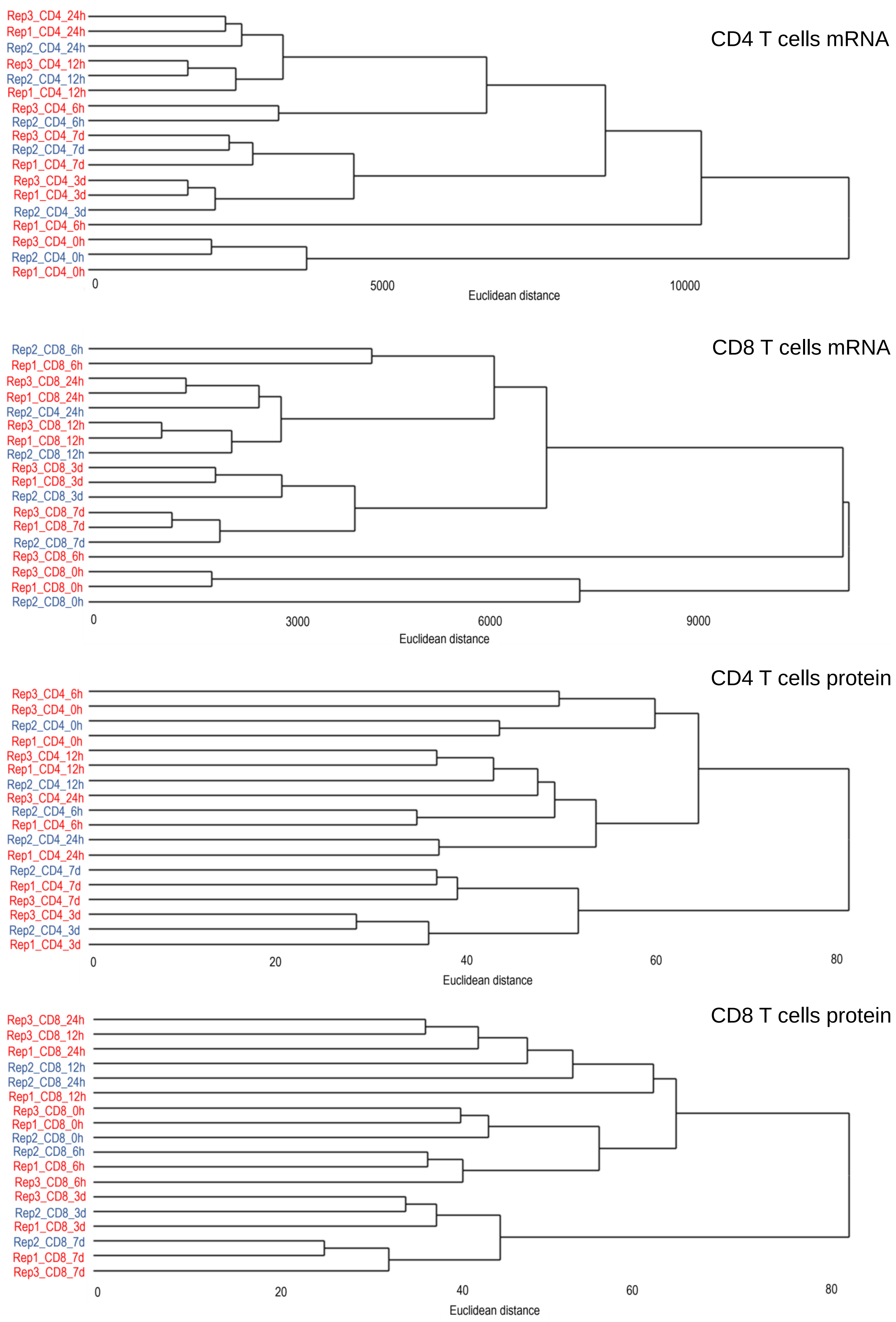

Column dendrograms showing the relationship of mRNA/protein expression data of CD4 and CD8 T cells among three biological replicates (Replicate 1 and 3 – females, in red Replicate 2 – male, in blue). mRNA transcripts and protein expression data of replicates were clustered using average linkage methods while Euclidean distance was used as the distance measurement method.

Supplementary Figure 4

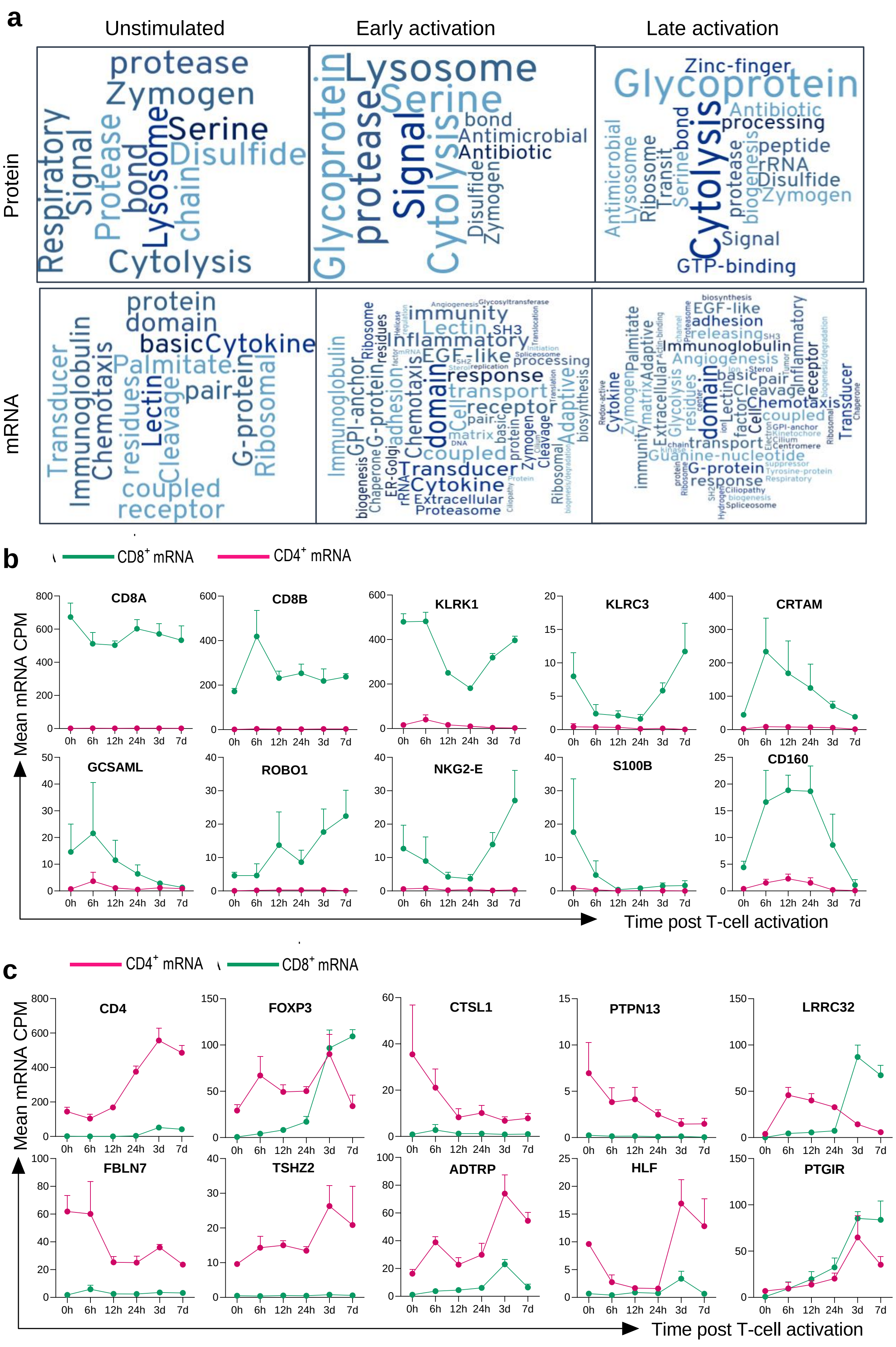

a. Word clouds showing the significant annotated UniProt key words revealed in functional enrichment analysis (FDR < 0.05) of differentially expressed mRNA and protein in CD8 versus CD4 T cells. Early activation word cloud represents annotated UniProt key words identified for 6h, 12h and 24h while late activation represents annotated UniProt key words identified for 3d and 7d.

b. Expression kinetics of the transcripts upregulated in CD4 T cells at 0h. mRNA expression values of each activation time point are given as mean and the standard mean of error (SEM) error bars.

c. Expression kinetics of the transcripts upregulated in CD8 T cells at 0h. mRNA expression values of each activation time point are given as mean and SEM error bars.

Supplementary Figure 5

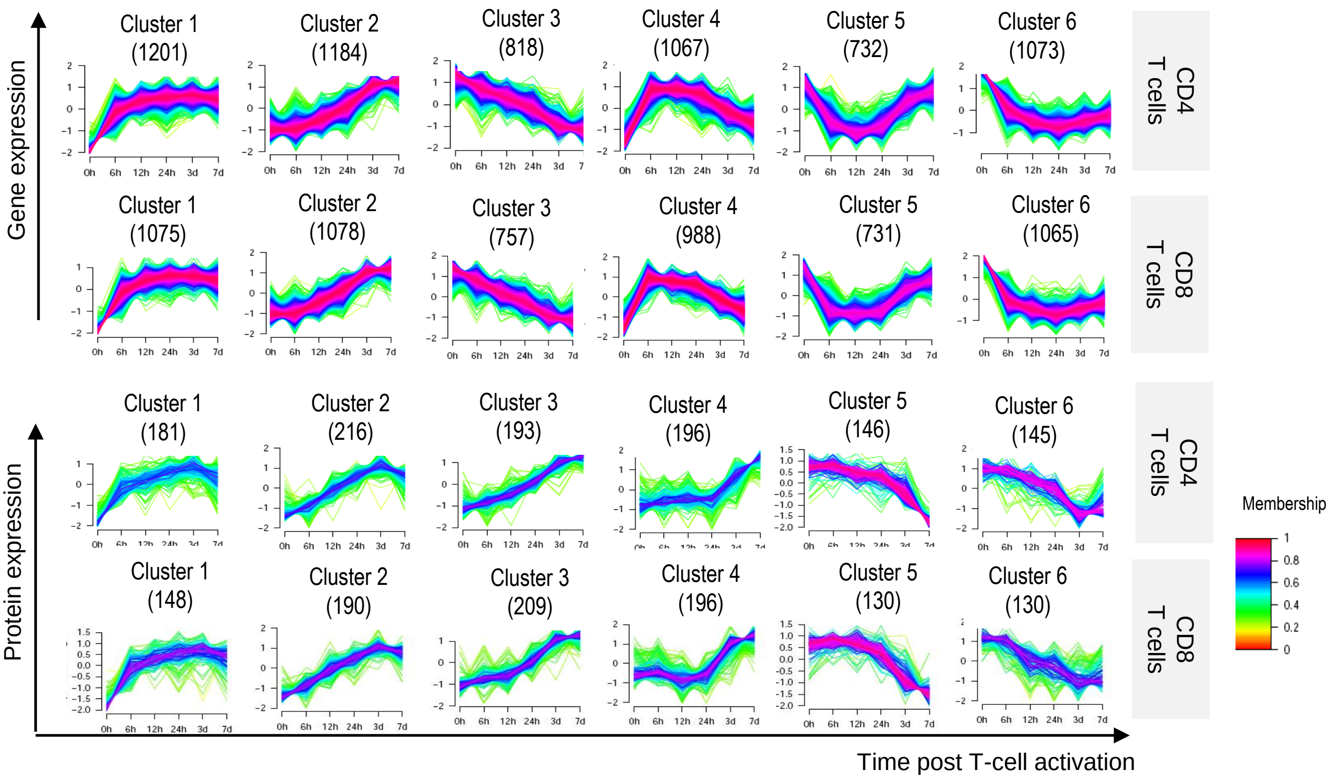

Expression of proteins and mRNA transcripts in each cluster is shown. mFuzz soft clustering was used to identify the co-expression clusters. Each line represents a mRNA/protein and the color indicates the membership of each mRNA/protein to the cluster.

Supplementary Figure 6

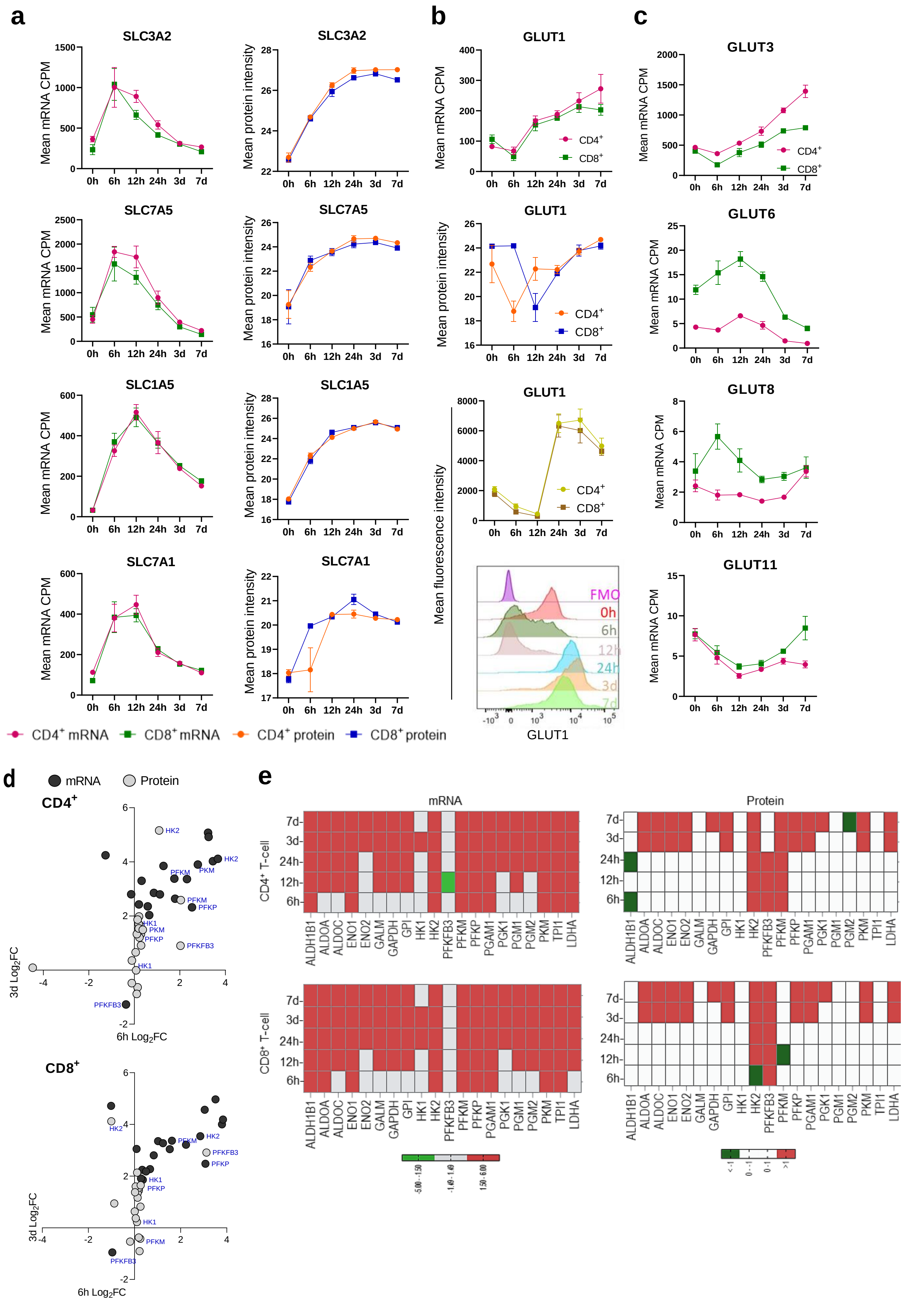

a. Expression kinetics of the amino acid transporters SLC3A2, SLC3A5, SLC1A5 and SLC7A1 at both mRNA and protein levels during CD4 and CD8 T cell activation. Expression values of each time point are given as mean and the standard mean of error (SEM) error bars.

b. Expression kinetics of the glucose transporter 1 (GLUT1) over the course of $CD4^+$ and $CD8^+$ T-cell activation. Data show values obtained from transcriptomics (mean mRNA CPM) and proteomics (protein intensity). In parallel, MFI (mean fluorescence intensity) values for cell surface GLUT1 were generated based on flow cytometric analysis and are shown alongside representative histograms of GLUT1 expression on CD4 T cells. Expression values of each time point are given as mean and the standard mean of error (SEM) error bars.

c. Expression kinetics of the glucose transporters 3, 6, 8 and 11 over the course of CD4 and CD8 T cell activation. Data show values obtained from transcriptomics (mRNA CPM). Expression values of each time point are given as mean and the standard mean of error (SEM) error bars.

d. Expression of mRNA transcripts and proteins in the glycolytic pathway. Data are given for both CD4 and CD8 T cells and represent enzymes and allosteric regulators regulated at 6 hours (y-axis) and/or 3d (x-axis) post-activation.

e. Heatmap representing the expression pattern of mRNA transcripts and proteins in the glycolytic pathway over the entire time course of CD4 and CD8 T cell activation.
